# Simultaneous capture and sequential detection of two malarial biomarkers on magnetic microparticles

**DOI:** 10.1016/j.talanta.2016.08.078

**Published:** 2016-12-01

**Authors:** Christine F. Markwalter, Keersten M. Ricks, Anna L. Bitting, Lwiindi Mudenda, David W. Wright

**Affiliations:** Department of Chemistry, Vanderbilt University, Station B 351822, Nashville, TN 37235, USA

**Keywords:** Immunoassay, Malaria, *Plasmodium* lactate dehydrogenase, *Plasmodium falciparum* histidine-rich protein II

## Abstract

We have developed a rapid magnetic microparticle-based detection strategy for malarial biomarkers *Plasmodium* lactate dehydrogenase (*p*LDH) and *Plasmodium falciparum* histidine-rich protein II (*Pf*HRPII). In this assay, magnetic particles functionalized with antibodies specific for *p*LDH and *Pf*HRPII as well as detection antibodies with distinct enzymes for each biomarker are added to parasitized lysed blood samples. Sandwich complexes for *p*LDH and *Pf*HRPII form on the surface of the magnetic beads, which are washed and sequentially re-suspended in detection enzyme substrate for each antigen. The developed simultaneous capture and sequential detection (SCSD) assay detects both biomarkers in samples as low as 2.0 parasites/µl, an order of magnitude below commercially available ELISA kits, has a total incubation time of 35 min, and was found to be reproducible between users over time. This assay provides a simple and efficient alternative to traditional 96-well plate ELISAs, which take 5–8 h to complete and are limited to one analyte. Further, the modularity of the magnetic bead-based SCSD ELISA format could serve as a platform for application to other diseases for which multi-biomarker detection is advantageous.

## Highlights

• Rapid, magnetic microparticle-based detection of *p*LDH and *Pf*HRPII from one sample.
• Detection of both biomarkers is critical in the context of malaria elimination.
• Total incubation time of 35 min.
• LODs an order of magnitude below commercial ELISA kits, within asymptomatic regime.
• Reproducible across users over time, and simple enough for novice users.

## Graphical abstract

Magnetic microparticle-based immunoassay reproducibly detects two malarial biomarkers in under an hour with detection limits an order of magnitude better than commercially available kits.

**Figure:**
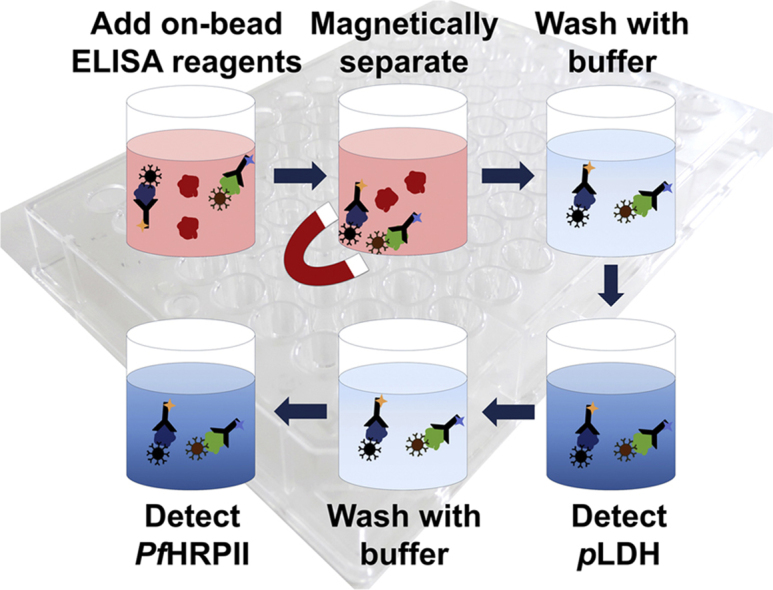

## 1 Introduction

Enzyme-linked immunosorbent assays (ELISAs) are the gold standard laboratory technique for quantitative and qualitative protein detection, serving as both powerful research tools and clinical diagnostics. These highly sensitive assays are typically performed in a microtiter plate, utilizing surface-bound antigen or antibody to bind a protein analyte and enzyme-conjugated, target-specific antibodies for detection. Although traditional singleplex ELISAs are laboratory “workhorses” for sensitive and specific protein detection, they require 5–8 h for completion and several incubation steps to ultimately develop signal. Further, conventional ELISAs are limited to detecting just one analyte from a single sample.

While traditional ELISAs are useful for diagnoses arising from one biomarker, diseases requiring multi-analyte detection to identify or inform treatment have led to the development of multiplexed immunoassays. A multiplexed immunoassay utilizes the same “sandwich” format (capture antibody, sample, detection antibody) as a conventional singleplex ELISA, except the former usually adopts fluorescent or chemiluminescent reporter systems rather than amplification of a colorimetric substrate by enzymes [1]. Two common formats for multiplexed immunoassays include planar arrays and bead-based suspension assays [2]. In typical, commercially available planar arrays (Quansys, MSD®), microliter volumes of capture antibodies for multiple protein biomarkers are printed discretely onto two-dimensional supports, such as slides or microtiter plates, using a high-resolution printer. The functionalized supports are then treated with sample, followed by reporter-labeled antibody. Signal is detected using a high-resolution scanner or fluorescence microscope [1]. In addition to multiplexing capabilities, planar micro-array immunoassays benefit from ambient analyte theory. According to ambient analyte theory, reducing the concentration of capture antibodies results in increased antibody binding site occupancy and thus higher assay sensitivity [3], [4]. However, these benefits are often off-set by mass transport limitations [1].

In contrast to planar arrays, bead-based suspension immunoassays are advantageous because they overcome mass transport limitations via active mixing throughout the liquid sample [5]. In a typical bead-based suspension immunoassay (Luminex™, Bio-PlexPro™, Cytometric Bead Arrays), fluorescent microbeads are functionalized with capture antibodies, mixed with a sample, and subsequently mixed with fluorescently-tagged detection antibodies, which allow for analyte detection via flow cytometric methods. Multiplexing capabilities arise when target-specific antibodies are functionalized to microbeads with varying fluorescent signatures distinguishable by flow cytometry [6].

There are several disadvantages to current multiplexed immunoassays. Both planar and bead-based immunoassays require laboratory infrastructure beyond that needed to perform singleplex conventional ELISAs; planar micro-array assays require high-resolution fluorescence scanners, and bead-based immunoassays require flow cytometric instrumentation for detection [1]. Further, planar micro-arrays require several addition, wash, and incubation steps totaling up to 3 h [7]. Commercially available bead-based suspension assays often require 3–4 h for completion, up to 1 h dedicated to the detection step [8]. To address these pitfalls, we have developed a magnetic bead-based ELISA in which two biomarkers are simultaneously captured and sequentially detected in less than 1 h with no laboratory infrastructure beyond what is required to perform a conventional singleplex well-plate ELISA.

We applied the developed magnetic bead-based ELISA to the detection of two malarial biomarkers: (1) *Plasmodium* lactate dehydrogenase (*p*LDH), and (2) *Plasmodium falciparum* histidine-rich protein II (*Pf*HRPII). Malaria, a mosquito-borne infectious disease caused by *Plasmodium* protozoan parasites, claimed over 400,000 lives in 2015 [9]. Accurate diagnosis of malaria is imperative for defining disease prevalence and distribution as well as monitoring impact of interventions. Furthermore, identification and proper treatment of asymptomatic cases (<200 parasites/µl), which serve as transmission reservoirs, are critical for eliminating the disease [10]. An assay that detects both *p*LDH and *Pf*HRPII is beneficial for several reasons. First, *p*LDH is a parasite metabolic enzyme, so it is present for infections resulting from any of the five species of malaria known to infect humans, whereas *Pf*HRPII is only present in *P. falciparum* infections [11], [12]. Thus, an assay that detects both biomarkers can differentiate between *P. falciparum* and non-*falciparum* infections, a distinction that determines proper treatment [13]. Second, *Pf*HRPII remains in host circulation for up to one month, whereas *p*LDH is known to clear within 24 h post parasite clearance, so a dual assay can distinguish resolved and active *P. falciparum* infections [14]. The magnetic bead-based simultaneous capture and sequential detection (SCSD) ELISA for *p*LDH and *Pf*HRPII would not only inform patient management, but also allow for more efficient and sensitive *P. falciparum* and non-*falciparum* epidemiology and transmission studies. The presented assay design is modular and can be applied to any set of two biomarkers provided validated antibody pairs are available.

## 2 Experimental

### 2.1 Reagents and materials

Dynabeads® MyOneTM Streptavidin T1 beads were purchased from Life Technologies (Cat #65601). Recombinant *P. falciparum* lactate dehydrogenase (rc*Pf*LDH) and recombinant *Plasmodium vivax* lactate dehydrogenase (rc*Pv*LDH) were purchased from CTK Biotech (Cat #A3005, #A3004). *P. falciparum* D6 strain was cultured in the lab. *P. falciparum* W2, Benin 1, and PH1 reference strains were obtained from the Foundation for Innovative New Diagnostics (FIND). Anti-*Pf*HRPII capture and detection antibodies were purchased from Abcam (ab9203 and ab30384). Pan-specific α-*p*LDH antibodies were purchased from AccessBio, Fitzgerald, and Vista Diagnostics (Table S1). BluePhos® Microwell Phosphatase substrate was purchased from KPL (#50-88-02), and TMB One was purchased from Promega (G7431). The ELISA kit for *p*LDH was purchased from SD Bioline, S. Korea (05EK40), and the ELISA kit for *Pf*HRPII was purchased from Cellabs, Australia (KM2).

### 2.2 *p*LDH antibody pair screen

Capture and detection antibodies were screened for use in the *p*LDH on-bead ELISA. Briefly, 64 antibody pairs were tested (8×8 matrix) in a checkerboard 96-well plate ELISA format. Each of the 8 antibodies was conjugated to alkaline phosphatase (AP) for detection (Abcam, ab102850). 100-µl solutions of 1 µg/ml unmodified anti-*p*LDH IgG were incubated for one hour in Immulon 2 HB 96-well plates (Thermo Scientific #3455). The plates were then washed 3 times with 1 × phosphate buffered saline (PBS) containing 0.1% Tween-20 (PBST). Next, 250 µl of 5% w/v bovine serum albumin (Fisher BP1600) in PBST was incubated for 2 h in each well. The plates were then washed 3 times with PBST. Samples consisting of 0 and 100 parasites/µl *P. falciparum* D6 culture or 0 and 500 pM rc*Pv*LDH were added to the plates in triplicate in PBST containing 0.1% BSA and incubated for 2 h. The plate was then washed 5 times with 1 × tris buffered saline (TBS) containing 0.1% Tween-20 (TBST). Next, 100 µl of 0.5 µg/ml of detection antibodies in TBST with 0.5% BSA was added to each well, and the plates were incubated for 1 h while protected from light. The plates were then washed 5 times with TBST, and 100 µl of BluePhos® Microwell Phosphatase Substrate was added to each well and incubated for 20 min while protected from light. The absorbance was measured at 620 nm using a Synergy H4 microplate reader. Signal-to-noise ratios were determined for each pair and antigen.

### 2.3 Blood sample preparation

Pooled human whole blood (Bioreclamation IVT, HMWBCPD) was spiked with D6 *P. falciparum* culture (at 18,450 parasites/µl) to the desired parasitemia. An equal volume of 2 × lysis buffer (100 mM potassium phosphate pH 8.0, 600 mM NaCl, 250 mM imidazole, 2% Triton-X-100) was then added, and the lysed blood was filtered through glass wool in a plastic syringe.

### 2.4 Preparation of mAb-functionalized magnetic beads

Target-specific antibody-functionalized beads were prepared as reported previously [15]. Briefly, α-*p*LDH (Vista, 19g7) or α-*Pf*HRPII (Abcam, ab9203) antibodies were biotinylated with EZ-Link NHS-PEG4-Biotin, No-Weigh Format (Thermo Pierce #21329) in PBS with a 20× excess of NHS-PEG4 Biotin. Remaining NHS-PEG4 biotin was removed using Zebra Spin Desalting Columns with a 7 K molecular weight cut-off (Thermo Pierce #89882). Next, 5 mg of Dynabeads® MyOne™ Streptavidin T1 was washed 3 times with PBS before incubating for 30 min with 500 µl of 0.4 mg/ml of biotinylated antibody in PBS. The beads were then washed 3 times with PBS and blocked with excess D-biotin in PBS for 30 min. Finally, the beads were washed 3 times and re-suspended in 500 µl of PBS with 0.01% Tween-20.

### 2.5 On-bead ELISA for *p*LDH

Solutions (200 µl) of parasitized lysed whole blood were placed in a Fisherbrand Flat-bottom PS 96-well plate (#12565501). Four µl HAMA blocker (Fitzgerald 85 R-1001), 10 µl of α-*p*LDH (19g7) magnetic beads, and 1.57 µl of 1201:AP (1.27 mg/ml) were added to each well and incubated on an orbital shaker for 15 min. Using a MagWell™ Magnetic Separator (EdgeBio #57624), the beads were separated from the supernatant and washed with 200 µl PBST. As a second wash, 100 µl PBST was added to the beads, which were then moved to new wells. Next, 100 µl BluePhos® Microwell Phosphatase Substrate was added to each well containing beads, and the plate was incubated for 15 min while protected from light. The supernatant was removed, and signal was measured by absorbance (620 nm) on a plate reader.

### 2.6 On-bead ELISA for *Pf*HRPII

Parasitized lysed whole blood solutions (200 µl) were placed in a Fisherbrand Flat-bottom PS 96-well plate. Four µl HAMA blocker, 5 µl of α-*Pf*HRPII (ab9203) magnetic beads, and 2 µl of MPFG-55P (0.1 mg/ml) were added to each well and incubated on an orbital shaker for 15 min. Using a MagWell™ Magnetic Separator, the beads were separated from the supernatant and washed with 200 µl PBST. As a second wash, 100 µl PBST were added to the beads, which were then moved to new wells. Next, 100 µl TMB One was added to each well containing beads, and the plate was incubated for 5 min while protected from light. The supernatant was removed, and the reaction was stopped with 100 µl of 2 M H_2_SO_4_. Signal was measured by absorbance (450 nm) on a plate reader.

### 2.7 On-bead simultaneous capture and sequential detection (SCSD) ELISA for *p*LDH and *Pf*HRPII

**Fig. 1:**
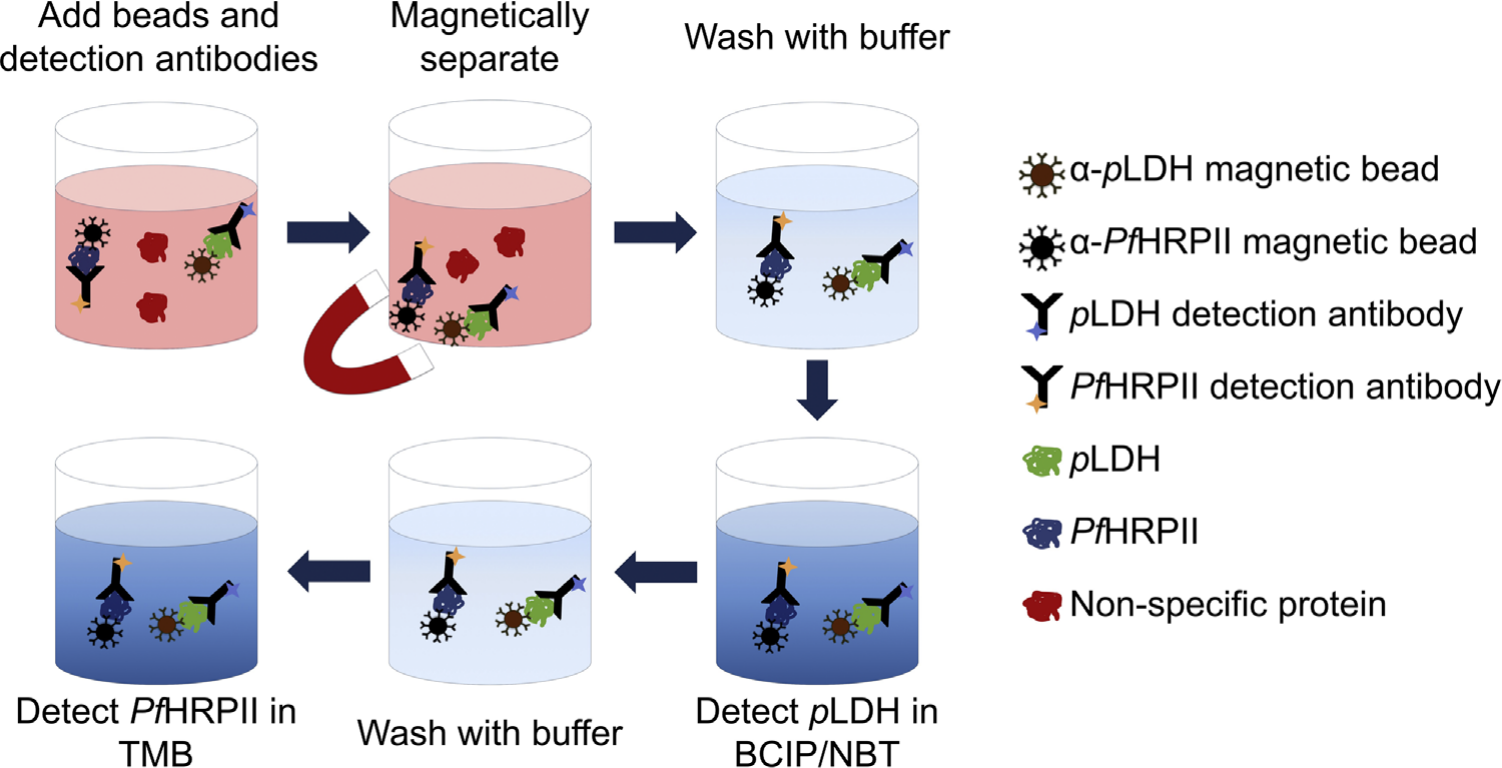
Workflow for on-bead SCSD ELISA for *p*LDH and *Pf*HRPII.

Solutions (200 µl) of parasitized lysed whole blood were placed in a Fisherbrand Flat-bottom PS 96-well plate. Four µl of HAMA blocking reagent, 10 µl of 19g7-conjugated magnetic beads, 5 µl of ab9203-conjugated magnetic beads, 1.57 µl of 1201:AP (1.27 mg/ml), and 2 µl of ab30384 (0.1 mg/ml) were added to each well and incubated on an orbital shaker for 15 min. The beads were pulled to the sides of the wells using a MagWell™ Magnetic Separator, and the supernatant was removed. The beads were washed with 200 µl PBST. As a second wash, 100 µl PBST were added to the beads, which were then moved to new wells. Next, 100 µl of BluePhos® Microwell Phosphatase Substrate was added to each well and incubated for 15 min while protected from light. The supernatant was removed and absorbance was measured at 620 nm (*p*LDH detection). The beads were then washed three times with PBST and moved to new wells on the third wash. Next, the beads were re-suspended in 100 µl of TMB One Solution and incubated for 5 min while protected from light. Finally, the supernatant was removed, and the reaction was quenched with 100 µl 2 M H_2_SO_4_ before absorbance was measured at 450 nm for detection of *Pf*HRPII. For both biomarkers, path length-corrected absorbance vs. concentration was plotted, and limits of detections (LODs) were calculated as the concentration at the minimum detectable signal (3*SD*_*blank*_+*s*_*blank*_). See Fig. 1 for on-bead SCSD ELISA workflow.

### 2.8 Validation

Intra-assay variation for the developed assays was determined by repeating standard curve measurements in triplicate (singleplex assays) or sextuplicate (SCSD assay) on the same plate (one user). The intra-assay variation (%CV) was found by taking the average relative standard deviation (RSD) of each repeated measurement. Inter-assay variation was determined by measuring standard curves in triplicate (singleplex assays) or sextuplicate (SCSD assay) over 5 days (one user). The inter-assay variation (%CV) was calculated by dividing the standard deviation of all absorbance measurements at a given concentration over 5 days divided the mean absorbance value at that concentration over the 5 days. For establishing inter-user variation, two users performed standard curves in sextuplicate over 5 days. The inter-user variation (%CV) was calculated as the average percent difference between the mean values for both users across all replicates for all days. Finally, the simplicity of the SCSD assay was evaluated by providing 4 blinded samples (including a blank) to a novice user. The user was allowed two practice rounds before measuring the unknown samples via the on-bead SCSD ELISA for *p*LDH and *Pf*HRPII. Using a paired Student's T Test, novice absorbance values for these samples were compared to the expected values from standard curves generated by the inter-assay variation measurements.

## 3 Results and discussion

### 3.1 Design and optimization of on-bead ELISAs for *p*LDH and *Pf*RHPII

Selection of the best capture and detection antibody pairs is crucial for developing sensitive and specific immunoassays. For *Pf*HRPII assays, C1–13 (ab9203) capture and MPFG-55P, a detection antibody conjugated to horseradish peroxidase (HRPx), have been previously validated as an appropriate pair for ELISA formats [16]. Piper et al. performed extensive screening of *p*LDH antibody pairs for immunochromatographic assays on nitrocellulose membranes [17]. However, binding kinetics in a lateral flow assay format do not represent the same equilibrium kinetics found in ELISAs [18]. Thus, a comprehensive screening of antibody pairs was conducted to evaluate performance in an ELISA format.

**Fig. 2:**
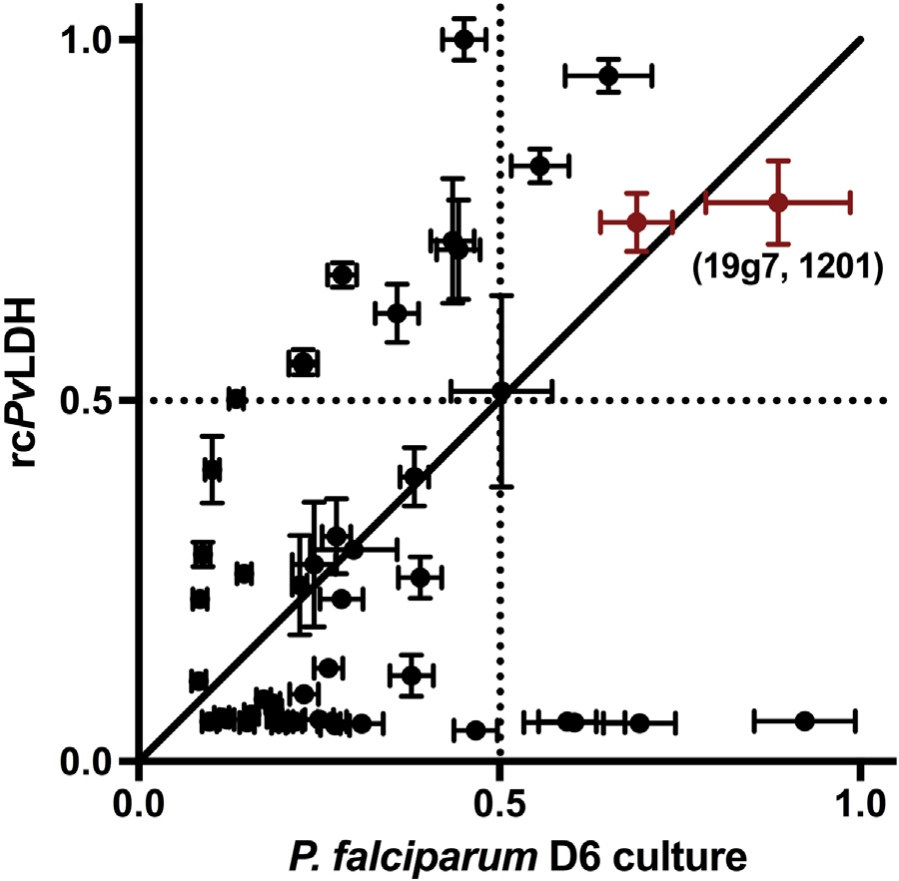
Pan-specific α-*p*LDH antibody pair screening results. Two candidate pairs are highlighted in red. 19g7 capture and 1201 detection antibodies were chosen for the *p*LDH on-bead ELISA.

In total, 64 antibody pairs were screened (8×8 matrix) in a checkerboard format. Each monoclonal antibody (Table S1) was conjugated to alkaline phosphatase for detection. All antibodies were immobilized in a polystyrene plate and allowed to bind to *Pf*LDH from *P. falciparum* D6 culture at 0 or 440 ± 40 pM or rc*Pv*LDH at 0 or 500 pM. Detection antibodies were then added to the plate, and signal was generated using a BCIP/NBT substrate. The resulting signal-to-noise ratio for each pair and for each antigen (Fig. S1) was normalized and plotted in Fig. 2. In this plot, each point represents one antibody pair, where the abscissa is the normalized signal-to-noise ratio for *Pf*LDH, and the ordinate is the normalized signal-to-noise ratio for rc*Pv*LDH. An ideal antibody pair would reside along the line y = x in the upper right-hand quadrant, indicating that it worked well for *p*LDH antigens from both species. Based on these criteria, two candidate pairs were identified and tested in an on-bead format (highlighted in red). Both highlighted pairs included 19g7 as a capture antibody and differed in detection antibodies (10-P09CS and 1201). Neither of these pairs were tested by Piper et al., nor were they sold as matched pairs from their respective manufacturer(s). However, Piper et al. did show that 19g7 worked well for several pairs as a capture antibody in a paper immunochromatographic assay format [17]. The pair with 19g7 capture and 1201:AP detection was chosen for the on-bead format, because it displayed lower background signal in the on-bead ELISA format.

With antibody pairs selected for *p*LDH and *Pf*HRPII, a one-step on-bead ELISA was developed and optimized for each individual biomarker. These individual assays were carried out by incubating magnetic beads functionalized with capture antibodies as well as enzyme-conjugated detection antibodies in samples consisting of 100 µl parasitized whole blood and 100 µl lysis buffer, allowing the sandwich complexes to form on the surface of the particles. The beads were then washed and re-suspended in the appropriate detection antibody substrate, and colorimetric signal was measured by absorbance. In order to maximize signal-to-noise ratios, several variables were optimized by varying the parameter of interest while holding all other assay parameters constant (Figs. S2 and S3). First, the amount of magnetic beads for biomarker capture was optimized to ensure there were enough binding sites to capture all the biomarker available, while minimizing nonspecific binding. Interestingly, for *Pf*HRPII, as the amount of C1–13-conjugated beads was increased beyond 50 µg, we observed a reduction in signal-to-noise ratio, likely due to the unique protein structure of *Pf*HRPII (Fig. S3**-**A). Because its secondary structure is simply a series of repeat-motifs, several capture antibodies may bind one *Pf*HRPII antigen, causing aggregation of the magnetic particles and preventing detection antibody from binding and producing signal [19]. In contrast, we do not see a significant decrease in signal-to-noise ratio for detection of *p*LDH when 19g7-conjugated beads are increased above saturation, since *p*LDH does not display repeated epitopes. Next, detection antibody concentration, sample incubation time, and substrate incubation time were optimized for each biomarker. Ideal conditions for the *p*LDH assay were found to be 100 µg 19g7-conjugated magnetic beads, 10 µg/ml 1201:AP detection antibody, 15-min sample incubation, and 15-min incubation in BCIP/NBT. For the *Pf*HRPII assay, 50 µg of C1–13-functionalized magnetic beads, 1 µg/ml MPFG-55P detection antibody, 15-min sample incubation, and 5-min incubation in TMB were chosen.

### 3.2 Performance of *p*LDH on-bead ELISA

**Fig. 3:**
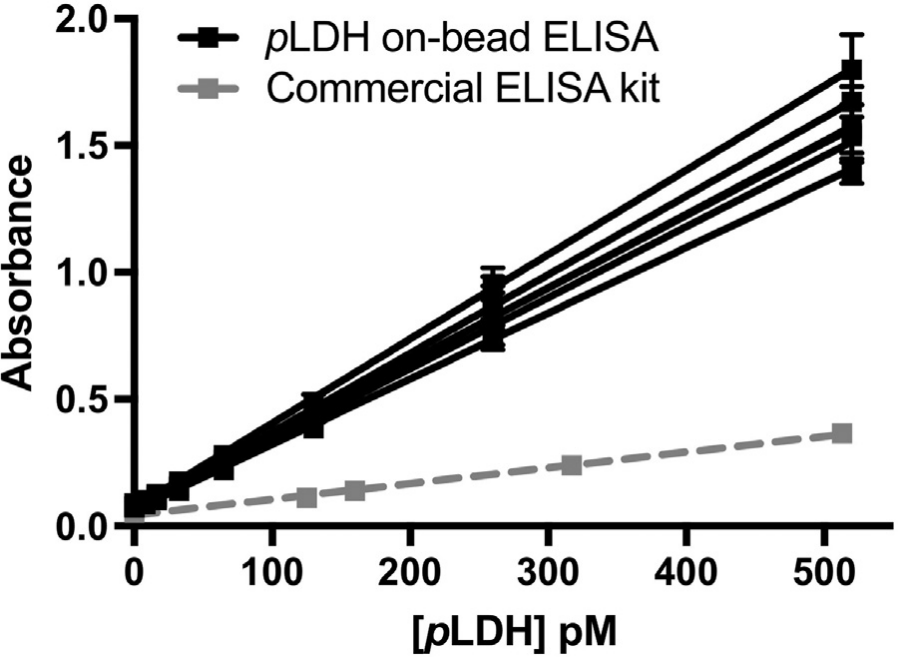
Standard curves for *p*LDH on-bead ELISA, measured at 620 nm, repeated in triplicate over 5 days (black) and SD Bioline Malaria Ag ELISA, measured at 450 nm (grey).

The *p*LDH on-bead ELISA was performed in lysed whole blood in triplicate, once per day over five days (Fig. 3). The linear range of the assay was found to be 7.0–520 pM *p*LDH. The intra-assay variation was 7.5%, and the inter-assay variation was 11%, below the acceptable biomedical assay variation values of 15% [20]. The limit of detection (LOD), defined by the concentration at which the signal is *s*_*blank*_ + 3*SD*_*blank*_, was 6.7 ± 3.4 pM, corresponding to about 5.2 parasites/µl of our in-house D6 *P. falciparum* culture, well within the asymptomatic regime. This LOD is three times lower than a commercially available well-plate ELISA kit for *p*LDH (Malaria Ag ELISA, SD Bioline, LOD = 19.3 ± 0.7 pM). Further, while the commercially available ELISA kit provides a pre-coated and blocked microtiter plate, it still required over 2 h of incubation time before results were generated. In contrast, our on-bead ELISA for *p*LDH, which is an order of magnitude more sensitive, is completed with a mere 30 min’ total incubation time.

### 3.3 Performance of *Pf*HRPII on-bead ELISA

**Fig. 4:**
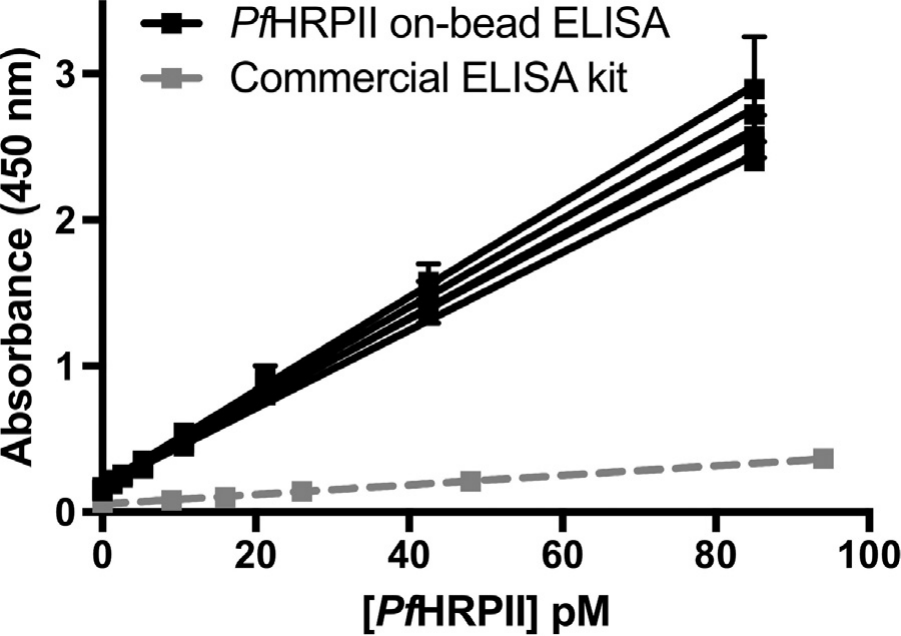
Standard curves for *Pf*HRPII on-bead ELISA repeated in triplicate over 5 days (black) and Cellabs Malaria Antigen (HRP2) CELISA (grey).

The *Pf*HRPII on-bead ELISA was evaluated in lysed whole blood in the same manner as the *p*LDH assay (Fig. 4). The linear range of the assay was found to be 1.0–85 pM *Pf*HRPII. The intra-assay variation was 4%, and the inter-assay variation was 7%, well below the acceptable value of 15% [20]. The LOD was 0.4±0.2 pM, corresponding to about 0.2 parasites/µl of our in-house D6 *P. falciparum* culture. This LOD for our 20-min *Pf*HRPII on-bead ELISA is over one order of magnitude lower than a 2.5-h commercially available well-plate ELISA kit for *Pf*HRPII (Malaria Antigen (HRP2) CELISA, Cellabs, LOD = 8.2 ± 0.2 pM).

### 3.4 Design of on-bead SCSD ELISA for *p*LDH and *Pf*HRPII

Fig. 1 shows the workflow for the on-bead SCSD ELISA. Before combining the two independent assays into the dual format, it was demonstrated that there was no cross-reactivity between *p*LDH and the *Pf*HRPII assay and vice versa (Fig. S4). To perform the SCSD assay, magnetic particles functionalized with capture antibodies for *p*LDH and *Pf*HRPII were incubated in lysed whole blood samples along with the detection antibodies for each biomarker. Throughout the assay, the beads for both biomarkers were processed and washed simultaneously. The optimized bead masses and detection antibody concentrations determined in the development of the individual assays were also used in the SCSD format. A sample incubation time of 15 min was chosen, since this time was found to be sufficient for sandwich complex formation in both the *p*LDH and *Pf*HRPII assays. The beads were then washed and re-suspended in BCIP/NBT for 15 min for the detection of *p*LDH via the AP-conjugated detection antibody. Absorbance of the supernatant was measured at 620 nm. It was found that AP detection must precede HRPx detection, due to the pH sensitivity of AP and the acidic nature of the HRPx substrate (Fig. S5). After *p*LDH detection, the beads were washed and re-suspended in TMB One solution for detection of *Pf*HRPII. The reaction was stopped, and *Pf*HRPII signal was measured by absorbance at 450 nm. In this work, the capture beads for the SCSD assay were prepared separately for each biomarker; however, to facilitate large-scale SCSD detection, a single batch of capture beads could be prepared using the proper 2:1 ratio of *p*LDH to *Pf*HRPII capture antibodies.

### 3.5 Performance of on-bead SCSD ELISA for *p*LDH and *Pf*HRPII

**Fig. 5:**
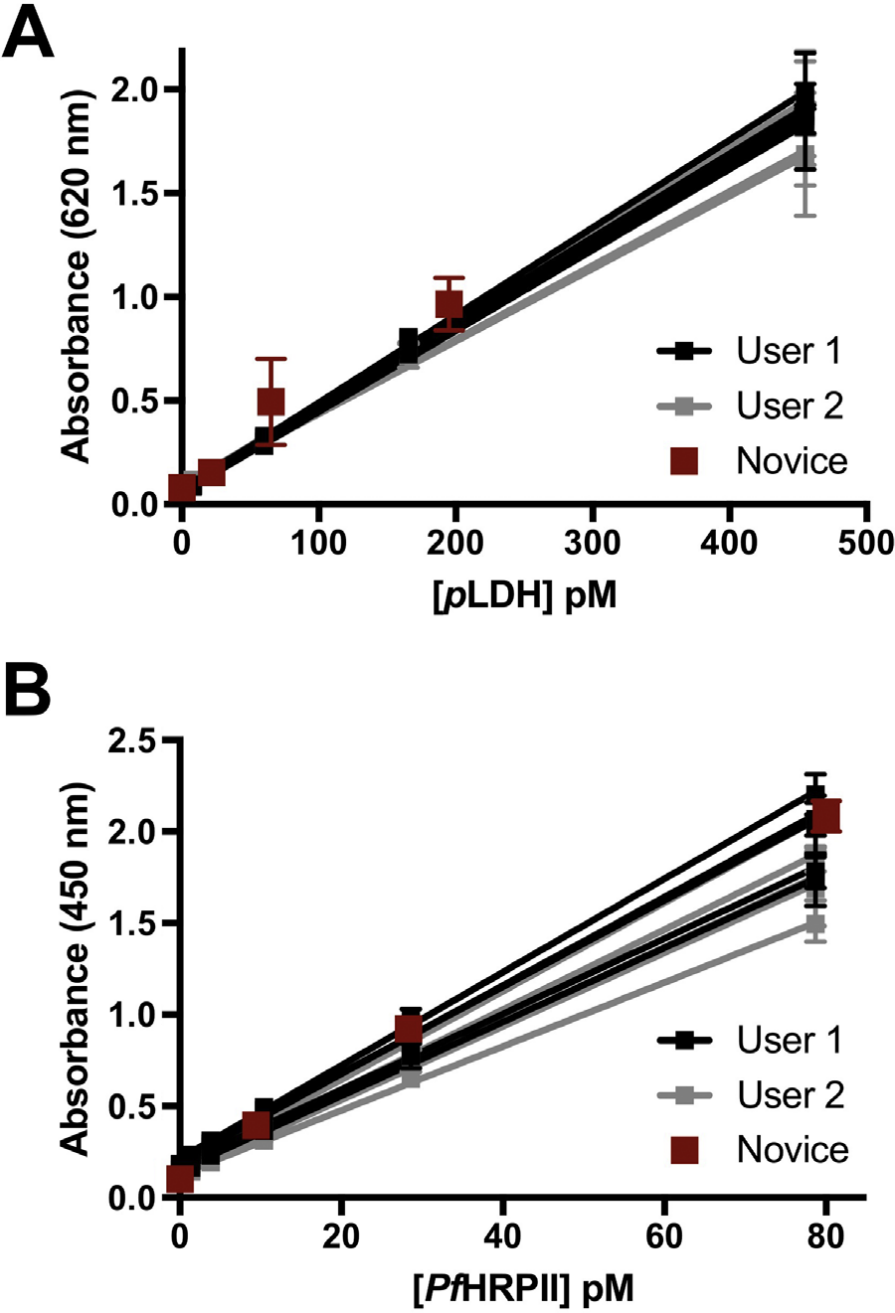
Two-user validation curves for on-bead SCSD ELISA for (A) *p*LDH and (B) *Pf*HRPII. Novice measurements are highlighted in red. (For interpretation of the references to color in this figure legend, the reader is referred to the web version of this article.)

The on-bead SCSD ELISA for *p*LDH and *Pf*HRPII was evaluated by two users measuring standard curves (n = 6) once per day over five days. A summary of the SCSD assay parameters for each biomarker is shown in Table 1. Linear ranges for each biomarker in the SCSD format were unchanged compared to the individual assays. The intra- and inter- assay variabilities for *p*LDH and *Pf*HRPII detection remained below the accepted value of 15%. The assay was reproducible between users for both biomarkers, with a 7.5% coefficient of variation for *p*LDH and a 20% coefficient of variation for *Pf*HRPII (Fig. 5). The simplicity of the assay was determined by providing a novice user blinded samples and comparing the signal obtained to that of an expert user. The novice measurements are highlighted in red in Fig. 5 and were not found to be significantly different from the expected absorbance values predicted by standard curves from expert measurements by paired T-tests (*p* = 0.1699 and *p* = 0.495 for *p*LDH and *Pf*HRPII, respectively). The LODs for *p*LDH and *Pf*HRPII were 2.6 ± 1.5 pM and 1.6 ± 1.0 pM, corresponding to 2.0 and 0.9 parasites/µl, respectively, for our in-house D6 *P. falciparum* culture. These detection limits remain an order of magnitude lower than those of commercially available ELISA kits for both biomarkers. Further, to detect both biomarkers using commercially available kits, two aliquots of sample would need to be processed in parallel for a total of more than 2 h before results are available. In contrast, the on-bead SCSD ELISA for *p*LDH and *Pf*HRPII measures both biomarkers from the same sample with incubation times totaling just 35 min.

The broad applicability of the on-bead SCSD ELISA was demonstrated by performing the assay on three additional *P. falciparum* strains. Standardized culture specimens (W2, Benin 1, and PH1 strains) designed for the development and evaluation of *Pf*HRPII diagnostics were obtained from the Foundation for Innovative New Diagnostics (FIND). These standards were received at a normalized concentration of 800 pg/ml *Pf*HRPII, corresponding to 14.4 pM and confirmed in our laboratory with the commercially available *Pf*HRPII CELISA kit. These samples were diluted 2-fold in human whole blood to a final concentration of 7.2 pM *Pf*HRPII before lysis, and an on-bead SCSD ELISA was performed in triplicate. The assay successfully detected both *p*LDH and *Pf*HRPII for all three strains tested, and the *Pf*HRPII concentrations obtained were not significantly different from the FIND reference values (Fig. S6). Because the assay performed well for multiple *P. falciparum* strains, and the *p*LDH portion of the assay was optimized for detection of the biomarker from both *P. falciparum* and *P. vivax*, we expect the on-bead SCSD ELISA to perform reliably with clinical samples.

Magnetic particle-based immunoassays have been developed previously for *p*LDH and *Pf*HRPII. For *p*LDH, magnetic microparticles were used to isolate *Plasmodium falciparum* lactate dehydrogenase (*Pf*LDH) from lysed whole blood. The biomarker was then detected using the Malstat assay, and enzymatic turnover assay catalyzed by *Pf*LDH [15]. The detection limit for this method was 26 pM *Pf*LDH, an order of magnitude higher than the developed on-bead SCSD ELISA for *p*LDH and *Pf*HRPII. Further, we have optimized the *p*LDH portion of the SCSD ELISA such that it can detect *p*LDH for both *P. falciparum* and *P. vivax*. For *Pf*HRPII, Castilho et al. developed an immunomagnetic detection strategy in which a sandwich complex was formed on the surface of magnetic micro- or nanoparticles [21]. Electrochemical and optical detection strategies were used, and the limit of detection was found to be 12 pM, an order of magnitude higher than the dual on-bead SCSD developed herein.

The developed on-bead SCSD ELISA is a rapid, simple, and sensitive method to quantitatively measure two biomarkers. The simultaneous capture aspect of the developed assay allows two biomarkers to be measured from one sample, reducing the volume of sample required (100 µl) to a single finger prick. The assay detection limits are lower than traditional well plate ELISAs, and the time-to-result is lower than currently available multiplexed immunoassays. Further, in contrast to commercially available multiplexed immunoassays, planar or suspension formats, the developed assay requires no laboratory equipment beyond what is required for conventional ELISAs. As such, it could be performed in settings where automated, hospital-grade diagnostic systems are impossible to implement due to lack of financial resources or infrastructure, efficiently providing accurate results with more clinical utility than traditional single-biomarker ELISAs. In the context of malaria elimination, the rapid and accurate detection of *p*LDH and *Pf*HRPII using our on-bead SCSD ELISA would be useful for several applications. For case management, the *p*LDH portion of the assay determines whether or not a patient has an active malaria infection, and the *Pf*HRPII portion distinguishes between *P. falciparum* and non-*falciparum* infections. Additionally, the LODs of the developed assay are well within the asymptomatic regime, allowing for detection and treatment of asymptomatic infections that contribute to the malaria transmission reservoir and would have otherwise been missed by commercially available ELISA kits or rapid diagnostic tests [10], [22]. Finally, our assay would be advantageous in the context of surveillance and intervention management, allowing rapid and sensitive measurement of disease distribution and trends for both *P. falciparum* and non-*falciparum* malaria.

While this work focused on detection of malarial biomarkers, the format of the developed on-bead SCSD ELISA could be generalized to any disease for which the detection of two biomarkers is advantageous. As long as a validated pair of antibodies is available or can be found for each biomarker and cross-reactivity between the two biomarkers is at a minimum, an on-bead SCSD ELISA can be developed and optimized.

## 4 Conclusions

We have developed a magnetic bead-based ELISA for the detection of *p*LDH and *Pf*HRPII in which sandwich complexes form on the surface of the magnetic beads directly in lysed whole blood samples. The biomarkers are detected sequentially in the appropriate detection enzyme substrates, with detection limits of 2.6 ± 1.5 pM for *p*LDH and 1.6 ± 1.0 pM for *Pf*HRPII, an order of magnitude better than commercially available ELISA kits for both biomarkers and within the asymptomatic regime for malaria. The low detection limits and high sensitivity of the assay can be attributed to active mixing of the beads within the sample to avoid mass transport limitations as well as careful assay optimization. The on-bead SCSD ELISA is repeatable and reproducible across multiple days and multiple users, and it is simple enough for novice users to produce accurate results. As such, it would be a valuable tool for case management and disease surveillance in the context of malaria elimination. Further, the developed on-bead SCSD ELISA format could be applied to any disease in which the detection of two biomarkers is beneficial, provided that antibody pairs are available for both biomarkers of interest.

## Figures and Tables

**Table 1:** Performance of on-bead SCSD ELISA for *p*LDH and *Pf*HRPII.

Parameter	*p*LDH	*Pf*HRPII
Linear range	7 – 500 pM	1.5 – 80 pM
LOD	2.6 ± 1.5 pM	1.6 ± 1.0 pM
Intra-assay variability	3.9%	6.2%
Inter-assay variability	6.4%	12.6%
Inter-user variability	7.5%	20%
