# Supplementary material for: Simultaneous capture and sequential detection of two malarial biomarkers on magnetic microparticles
Source: Talanta. 2016 Dec 1;161:443–9. doi: 10.1016/j.talanta.2016.08.078 (PMC5080451; doi:10.1016/j.talanta.2016.08.078)
Supplement: Supplementary file 1 — Supplementary material [file mmc1.docx]

**Simultaneous capture and sequential detection of two malarial biomarkers on magnetic microparticles**

Christine F. Markwalter,^#^ Keersten M. Ricks,^#1^ Anna L. Bitting, Lwiindi Mudenda, David W. Wright*

Department of Chemistry, Vanderbilt University, Station B 351822, Nashville, TN, 37235, USA

Fax: 615-343-1234

Tel: 615-322-2636

*Email address: david.wright@vanderbilt.edu

^1^Present Address: US Army Medical Research Institute of Infectious Diseases, Fort Detrick, MD

#Authors contributed equally to this work

Table of Contents

| Pan-specific *p*LDH antibody clones and sources.................................................................... | S-2 |
| --- | --- |
| Checkerboard ELISA results for *p*LDH antibodies................................................................ | S-3 |
| *p*LDH on-bead ELISA optimization results............................................................................ | S-4 |
| *Pf*HRPII on-bead ELISA optimization results........................................................................ | S-5 |
| Assessment of cross-reactivity of *p*LDH and *Pf*HRPII on-bead ELISAs............................... | S-6 |
| Detection order for on-bead SCSD ELISA for *p*LDH and *Pf*HRPII...................................... | S-7 |
| On-bead SCSD ELISA for multiple *P. falciparum* strains..................................................... | S-8 |

**Table S1**. Pan-specific *p*LDH antibodies screened for ELISA activity

| Source | Clone |
| --- | --- |
| AccessBio | 14c2 |
| Vista | 6c9 |
|  | 12g1 |
|  | 19g7 |
|  | 1201 |
|  | 1246 |
| Fitzgerald | 10-P09CS |
|  | 10-P09F |

**Figure S1**. Signal-to-noise ratios for 64 antibody pairs for 100 parasites/µl (A) *P. falciparum* culture and (B) 500 pM recombinant *Pv*LDH.

**Figure S2**. Optimization of (A) bead mass, (B) detection antibody concentration, (C) sample incubation time, and (D) substrate incubation time for the *p*LDH on-bead ELISA.

**Figure S3**. Optimization of (A) bead mass, (B) detection antibody concentration, (C) sample incubation time, and (D) substrate incubation time for the *Pf*HRPII on-bead ELISA.

**Figure S4**. The (A) *p*LDH on-bead ELISA is not cross-reactive with recombinant *Pf*HRPII (*p* = 0.8304), and the (B) *Pf*HRPII on-bead ELISA does not react with recombinant *Pf*LDH (*p* = 0.7179) by two-way ANOVAs. The spiked concentrations (17 nM and 13 nM, respectively), represent a 20x excess of the biomarker concentration expected at 50 parasites/µl.

**Figure S5.** Signal to noise ratios for (A) *p*LDH and (B) *Pf*HRPII portions of the on-bead SCSD ELISA when the order of alkaline phosphatase (*p*LDH) and horseradish peroxidase (*Pf*HRPII) detection is switched. A significant decrease in signal-to-noise ratio for *p*LDH is observed when *Pf*HRPII is detected first. However, there is no significant difference for *Pf*HRPII detection in either order of detection. Thus, alkaline phosphatase must be detected first. (C) The hypothesis that the acidic nature of the TMB substrate deactivates alkaline phosphatase was tested by performing a *p*LDH on-bead ELISA in which the beads were incubated in citrate buffer pH 3.5 (same as Promega TMB One Solution) before detection. Signal for a 50 parasite/µl sample was not significantly different from the blank.

**Figure S6**. On-bead SCSD ELISA (A) *p*LDH and (B) *Pf*HRPII performance for W2, Benin 1, and PH1 strains as compared to reference values (for *Pf*HRPII) obtained from FIND.
